# The Potential Application and Promising Role of Targeted Therapy in Pulmonary Arterial Hypertension

**DOI:** 10.3390/biomedicines10061415

**Published:** 2022-06-15

**Authors:** Meng-Chien Willie Hsieh, Wei-Ting Wang, Jwu-Lai Yeh, Chuang-Yu Lin, Yur-Ren Kuo, Su-Shin Lee, Ming-Feng Hou, Yi-Chia Wu

**Affiliations:** 1 Division of Plastic Surgery, Department of Surgery, Kaohsiung Medical University Hospital, Kaohsiung 80708, Taiwan; mch21@alumni.duke.edu (M.-C.W.H.); damouguai@gmail.com (W.-T.W.); kuoyrren@gmail.com (Y.-R.K.); k831702000@gmail.com (S.-S.L.); 2 Department of Plastic Surgery, Kaohsiung Municipal Ta-Tung Hospital, Kaohsiung 80145, Taiwan; 3 Department of Pharmacology, School of Medicine, College of Medicine, Kaohsiung Medical University, Kaohsiung 80708, Taiwan; jwulai@kmu.edu.tw; 4 Department of Biomedical Science and Environmental Biology, College of Life Science, Kaohsiung Medical University, Kaohsiung 80708, Taiwan; lincy@kmu.edu.tw; 5 Department of Surgery, School of Medicine, Kaohsiung Medical University, Kaohsiung 80708, Taiwan; mifeho@kmu.edu.tw; 6 Regenerative Medicine and Cell Therapy Research Center, Kaohsiung Medical University, Kaohsiung 80708, Taiwan; 7 Department of Surgery, Kaohsiung Municipal Siaogang Hospital, Kaohsiung 81267, Taiwan; 8 Division of Breast Oncology and Surgery, Department of Surgery, Kaohsiung Medical University Hospital, Kaohsiung 80708, Taiwan

**Keywords:** pulmonary arterial hypertension, molecular base pathophysiology, targeted therapy, Warburg Effect

## Abstract

Pulmonary arterial hypertension (PAH) is a rare yet serious progressive disorder that is currently incurable. This female-predominant disease unfolds as a pan-vasculopathy that affects all layers of the vessel wall. Five classes of pharmacological agents currently exist to target the three major cellular signaling pathways identified in PAH but are incapable of effectively reversing the disease progression. While several targets have been identified for therapy, none of the current PAH specific therapies are curative and cost-effective as they fail to reverse vascular remodeling and do not address the cancer-like features of PAH. Our purpose is to review the current literature on the therapeutic management of PAH, as well as the molecular targets under consideration for therapy so as to shed light on the potential role and future promise of novel strategies in treating this high-mortality disease. This review study summarizes and discusses the potential therapeutic targets to be employed against PAH. In addition to the three major conventional pathways already used in PAH therapy, targeting PDGF/PDGFR signaling, regulators in glycolytic metabolism, PI3K/AKT pathways, mitochondrial heat shock protein 90 (HSP90), high-mobility group box-1 (HMGB1), and bromodomain and extra-terminal (BET) proteins by using their specific inhibitors, or a pharmacological induction of the p53 expression, could be attractive strategies for treating PAH.

## 1. Background

Pulmonary arterial hypertension (PAH) is a rare yet serious progressive disorder that induces right ventricular failure (RVF) and high mortality as a result of pulmonary microvasculature remodeling due to an increase in pulmonary vascular resistance (PVR) [1,2,3]. At the 6th World Symposium on Pulmonary Hypertension, PAH was redefined with the following hemodynamic criteria: a mean pulmonary artery pressure (mPAP) of >20 mmHg, pulmonary arterial wedge pressure of >15 mmHg, and pulmonary vascular resistance index of ≥3 Wood units (WU) [4]. In the classification of pulmonary hypertension (PH), five major groups have been identified based on the pathophysiological mechanism. Group 1 refers to pulmonary arterial hypertension and is an uncommon form of PH, which includes idiopathic PAH, heritable PAH, drug- and toxin-induced PAH, associated PAH (APAH), persistent PH of the newborn syndrome, etc. Group 2 describes the more common form of PH due to left heart disease. Group 3 is PH caused by lung diseases and/or hypoxia. Group 4 PH is caused by chronic thromboembolism and other pulmonary artery obstructions. For those not classifiable into the previous four categories, Group 5 encompasses PH with unclear and/or multifactorial mechanisms, such as hematological disorders, systemic and metabolic disorders, renal diseases, and complex congenital heart diseases [5].

The classic clinical symptoms and signs of PAH include fatigue, lethargy, dyspnea, exertional presyncope/syncope, chest pain, edema/ascites, and lip and skin cyanosis, depending on the severity of the pulmonary vascular abnormality and right ventricular dysfunction. As a rare disease, the prevalence of PAH is still estimated to be approximately 26–33/million in the US and 20–40/million in Europe [6]. PAH more commonly affects females with a female-to-male ratio ranging from 1.9:1 to 4.1:1, with the average age of diagnosis reported to be about 50 years [7].

Unfortunately, this life-threatening disease is currently incurable. Clinical approaches in PAH therapeutics merely rely on symptomatic relief, which can only temporarily delay the deterioration of the disease but are unable to completely cure the disease, especially in severe cases. We hereby review the current literature on the therapeutic management of PAH and try to shed light on the potential role and future promise of novel strategies in treating this high-mortality disease. The research methodology of this study was a literature review conducted by searching on the PubMed^®^ Database for publications using the keywords as provided.

## 2. The Etiology of PAH

### 2.1. Physiopathogenesis of PAH

PAH manifests as an endothelial dysfunction and a pan-vasculopathy that involves the entire vessel wall. Pathological abnormalities can be found in all layers of the pulmonary artery, from the proliferation of vascular endothelial cells in the intima and hypertrophy due to pulmonary artery smooth muscle cells (PASMCs) in the media to the senescence of fibroblast cells in the adventitia [8]. More specifically, the constrictive lesions characteristic of PAH are the combined results of intimal and adventitial thickening due to the proliferation and aggregation of collagen and connective tissue cells, which induce fibrosis [9]. Moreover, complement system components, autoantibodies, and inflammatory cells (neutrophils) in the vessel lumen also deposit in the endothelium to eventually infiltrate the medial layer [10]. These defects, especially with the hyperproliferation of PASMCs, contribute to inflammation, vasoconstriction, and fibrotic changes, which then decrease vascular compliance and, eventually, cause right ventricular failure as a consequence of an elevated right ventricular afterload [8].

### 2.2. The Molecular Basis of PAH Pathogenesis

**Figure 1:**
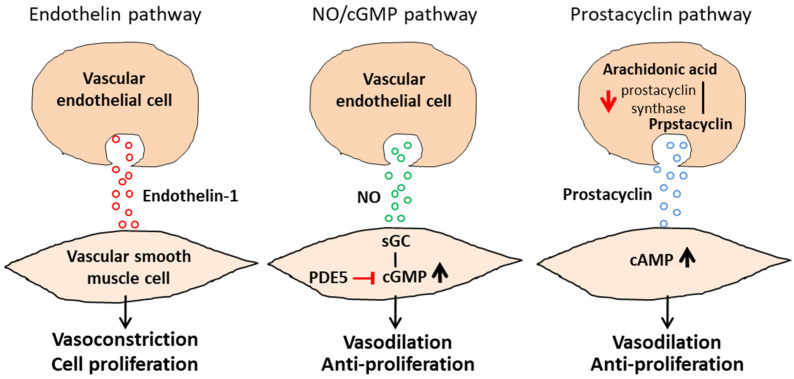
**Three conventional pathways in PAH pathogenesis.** In the endothelin pathway, vascular endothelial cells release endothelin-1, which induces vascular smooth muscle proliferation and vasoconstriction. In the NO/cGMP and prostacyclin pathway, the functional anti-proliferative and vasodilative ability of NO and prostacyclin signaling are hindered by PDE5 and prostacyclin synthase deficiency, respectively, which result in vascular smooth muscle proliferation and vasoconstriction, and further PAH pathogenesis. Red arrow: downregulated; Black arrow: upregulated.

Three major cellular signaling pathways have been identified in PAH: (1) the endothelin pathway, (2) the nitric oxide (NO)/cyclic guanosine, monophosphate (cGMP) pathway, and (3) the prostacyclin pathway [11], which are documented to be associated with the regulation of endothelial factors affecting vasoconstriction, vasodilation, proliferation, and mitogenesis [12]. The endothelin pathway involves vascular endothelial cells that secrete endothelin-1, which induces the vasoconstriction and proliferation of vascular smooth muscle cells. An increase in the circulating level of endothelin-1 and its expression in pulmonary vascularity have been implicated in PAH [12]. Additionally, nitric oxide (NO) is also considered a vasodilator in PAH treatment. In the NO/cGMP pathway, NO is a potent endogenous vasodilator that stimulates soluble guanylate cyclase (sGC) that, in turn, increases intracellular cGMP, which has both vasodilatory and antiproliferative effects on the smooth muscle of pulmonary vessels [13]. As a result, disruption in NO and natriuretic peptide signaling leads to a cGMP deficiency. In the pathogenesis of PAH, an increased expression of phosphodiesterase (PDE) type 5 can be observed, which is the main enzyme responsible for degrading cGMP within the pulmonary vascular smooth muscle [14]. In the prostacyclin pathway, the endothelial metabolism of arachidonic acid produces prostacyclin, which increases the production of cyclic adenosine monophosphate (cAMP) in order to relax the vascular smooth muscle to achieve vasodilation. The signaling of prostacyclin, which hinders the proliferation of endothelial cells and aggregation of platelets, can decrease intravascular thrombotic events [12]. In the pulmonary vascular endothelium of PAH patients, there is a decreased expression of the primary enzyme in charge of prostacyclin synthesis and prostacyclin synthase, thereby reducing prostacyclin circulating levels [15]. These three conventional pathways are displayed in Figure 1.

**Figure 2:**
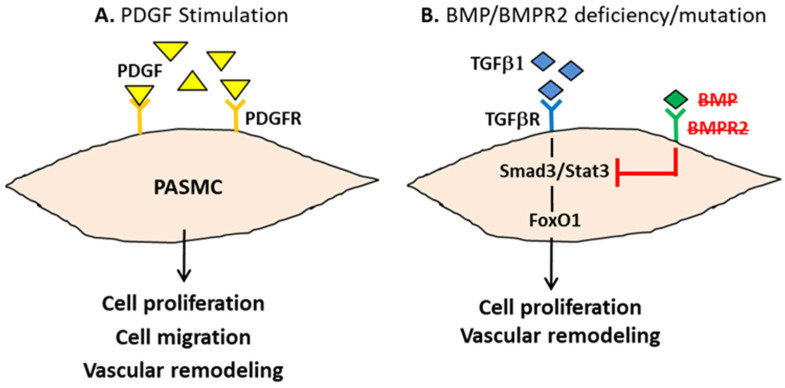
**The crucial roles of PDGF stimulation and BMP/BMPR2 aberration in PAH pathogenesis**. (**A**) PDGF binds to its receptor tyrosine kinase PDGFR to trigger cell proliferation and migration in PASMCs, which further leads to vascular remodeling. (**B**) BMP deficiency or BMPR2 mutation results in an impaired antagonistic activity to suppress TGF β1-Stat3-FoxO1 signaling-induced PASMCs proliferation and vascular remodeling.

During PAH pathogenesis, the platelet-derived growth factor (PDGF) has been identified as a major trigger of unregulated PASMCs’ proliferation and migration, leading to vascular remodeling via highly selective receptor tyrosine kinases [16]. The PDGF is a major mitogen, which consists of two structurally similar polypeptides (A and B chains) to form homo- or heterodimers. The PDGF stimulates its tyrosine kinase receptors, including the PDGFRα and the PDGFRβ, which further promotes the cell growth of the PASMCs and endothelial cells. Both the PDGF and PDGFR expressions have been confirmed to be increased in the pulmonary arteries of PAH patients (Figure 2A).

A reduction in the functional protein of the bone morphogenetic protein (BMP) and mutations of the BMP receptor 2 (BMPR2) have also been suggested to play a crucial role in PAH development. The BMP and BMPR2 serve as antagonists to transforming the growth factor β (TGFβ) pathways. The BMP2-BMPR2 counteracts the pro-proliferative TGFβ1 signaling by physically interacting with Smad3 and Stat3 to disturb the TGFβ1-Stat3-Forkhead box O1 (FoxO1) axis in PASMCs [17]. Since TGFβ1 signaling was found to be highly expressed in both experimental animal models and clinical PAH patients, therefore, deviant BMP signaling and epigenetic dysregulation promote cell proliferation, which dramatically increases the risk of PAH pathogenesis [18] (Figure 2B).

## 3. Similarities between PAH and Cancer

### 3.1. Warburg Effect 

The proliferative properties and mechanisms of PAH were first described to be cancer-like by Rubin Tuder in 1998. The irreversible microvascular remodeling of PAH consists of pulmonary arterial wall thickening caused by the uncontrolled proliferation, migration, and diminished apoptosis of PASMCs [19]. An abnormal metabolic alteration of pulmonary artery smooth muscle cells (PASMCs), called the Warburg Effect, has been described to resemble cancer as the cells are more inclined to enter the anaerobic respiratory pathway with upregulated glycolysis as opposed to mitochondrial respiration [20]. Proliferating PASMCs exhibit an upregulated glycolysis for the major source of adenosine triphosphate (ATP) production instead of suppressed mitochondrial glucose oxidation [21]. The upregulation of the pyruvate dehydrogenase kinase expression suppresses oxidative phosphorylation and inhibits pyruvate dehydrogenase (PDH) [22]. The cell metabolism then shifts to rely more on glycolysis, which accelerates proliferation and evades mitochondrial apoptosis. Moreover, PAH and cancer cells further achieve proliferation and oppose cell death signals by way of increasing fatty acid synthesis and glutamine metabolism [23].

### 3.2. Mitochondrial Dysfunction 

In the metabolism of healthy cells, mitochondria are constantly undergoing fusion and fission. However, mitochondrial fission and fragmentation are observed in PAH cells, which are patterns similarly seen in various cancer cells [24]. The ratio of dysfunctional mitochondria is increased in PAH cells, which leads to cellular toxicity [19]. While the Warburg metabolism is usually associated with the PASMCs of PAH patients, their pulmonary artery endothelial cells, adventitial fibroblasts, and even cardiomyocytes have also been observed to undergo similar metabolic alterations [18]. In preclinical studies, positron emission tomography even revealed an increase in fluorodeoxyglucose uptake in the right ventricle and lungs of PAH patients, which was attributed to the Warburg metabolism [25]. Dysregulated BMP signaling, due to the mutation of the BMPR2, has been demonstrated to be the reason for mitochondrial dysfunction in PAH development. The BMP2 serves as an inhibitory regulator of TGFβ1-induced mitochondrial respiration in PASMCs. Suppressing the BMPR2 in pulmonary artery endothelial cells induced mitochondrial fission and triggered enhanced glycolysis [26]. Despite the key difference between PAH from cancer cells, being the result of the excessive proliferation of endothelial cells, muscle cells, and fibroblasts, as opposed to cancer cells, the PASMCs in PAH pathogenesis and cancer cells share numerous similarities (Table 1).

## 4. Current Strategies in PAH Therapeutics

### 4.1. Conventional Treatment

The conventional treatment of PAH mostly relies on supportive measures, including supervised rehabilitation, birth control advice, and oxygen supplementation. Patient education on salt and fluid restriction is important in decreasing volume overload in the already compromised ventricular reserve [18]. Rehabilitation through exercise training is highly encouraged in PAH [27], often in the form of low-intensity aerobic exercises to avoid inducing syncope. When the oxygen saturation level drops below 90%, supplemental oxygen therapy should be provided to restore exercise capacity [28]. In light of the correlation between pregnancy and high mortality in PAH, birth control measures are strongly advised in women in the reproductive age group [29]. With hysteroscopic sterilization being the favored option for contraception, progesterone-only intrauterine devices, pills, and tubal ligation can also be considered [30]. However, contraceptives that contain estrogen and progesterone are contraindicated due to the elevated risk of thrombosis [30].

### 4.2. Background Therapies

In addition to providing supportive measures, background therapies can be implemented for the management of PAH-related symptoms and signs. With the effect of decreasing volume overload, diuretics are prescribed to alleviate venous congestion caused by RVF [31]. In spite of the short-term effects of increasing the right ventricular contractility and cardiac output seen in hemodynamic studies, digoxin has not yet been proven to be beneficial for long-term use in PAH [32]. Besides right ventricular dysfunction, vascular thrombosis can often be found in the small pulmonary vessels of PAH patients [33]. Anticoagulation therapy with warfarin has been demonstrated to improve the survival rate in several prospective and retrospective studies [34]. Long-term use of warfarin therapy has been recommended in IPAH, heritable PAH, or anorexigen-induced PAH patients to reach an international normalized ratio goal of 1.5–2.5 [35]. Nevertheless, some registry studies failed to prove the benefits of anticoagulation therapy, particularly in patients with associated pulmonary arterial hypertension (APAH) [36].

## 5. Targeted Therapies in PAH Therapeutics

### 5.1. Introduction

Targeted therapy is a type of treatment primarily employed in cancer therapy, which uses drugs or substances designed to precisely “target” specific proteins or genes that are abnormally altered in pathological aberrant cells during pathogenesis. This therapeutic modality also serves to influence various cell consequences, including the suppression of cell proliferation, migration, and/or the promotion of cell apoptosis. In recent times, the consideration of resorting to targeted therapy in PAH therapeutics has gained greater traction and garnered unprecedented attention.

### 5.2. Pharmacological Agents for the Three Major Cellular Signaling Pathways

Regarding PAH-specific therapies, there are five classes of pharmacological agents that target the three major cellular signaling pathways: (1) the endothelin pathway, (2) the NO/cGMP pathway, and (3) the prostacyclin pathway [11]. In the endothelin pathway, as endothelin-1 induces cell proliferation in vascular smooth muscle cells and further leads to vascular remodeling, a blockade of this pathway, using endothelin receptor antagonists, such as ambrisentan, bosentan, and macitentan, can induce pulmonary vasodilation. Similarly, endothelin receptor antagonists (Bosentan) non-selectively target endothelin receptors type A and B (ETA and ETB) or selectively target ETA receptors (Aambrisentan, Macitentan) to block this pathway, hence causing pulmonary vasodilation [10,35].

In the NO/cGMP pathway, PDE type 5 inhibitors (Tatalafil and Sildenafil) hamper the metabolic activity of cGMP so as to enhance the vasodilatory properties of NO and the natriuretic peptides, while sGC stimulators or activators (Riociguat) increase the activity of sGC and cGMP synthesis to cause smooth muscle relaxation and vasodilation [12]. From two of our previous studies, we also reported that using the glucagon-like peptide-1 receptor (GLP-1R) agonist, liraglutide, which is widely applied in diabetes therapeutics, could prevent and reverse monocrotaline (MCT)-induced PAH by suppressing ET-1 and enhancing the eNOS/sGC/PKG pathways [37]. Interestingly, liraglutide was also disclosed to ameliorate PAH by interfering with the Drp1/NOX- and Atg-5/Atg-7/Beclin-1/LC3β pathways, which are involved in autophagy [38].

In the prostacyclin pathway, the prostacyclin serves as a vasodilator with antithrombotic properties. The administration of a prostacyclin receptor agonist or a prostacyclin derivative rescues the decreased prostacyclin synthase and the reduced circulating prostacyclin in PAH and, thereby, is considered a strategy against PAH [11]. Both parenteral prostacyclin analogues (Epoprostenol, Treprostinil, and Iloprost) and the oral prostacyclin receptor agonist (Selexipag) serve to increase prostacyclin signaling [10,35].

### 5.3. Calcium-Channel Blockade

Specific therapy can also target a fourth relevant molecular pathway, namely the voltage-gated, L-type calcium channels. For idiopathic PAH (IPAH), calcium-channel blockers (CCB) proved to be effective in extending long-term survival [39]. CCBs (amlodipine, nifedipine, and diltiazem) are indicated only in PAH patients with a positive vasodilator test, who only represent 5–10% of all cases and can have a five-year survival rate of 90% with CCB monotherapy [40]. During therapy with CCBs, patients are closely monitored for its therapeutic effect and, if symptoms deteriorate, are timely switched to PAH-specific therapies [41].

### 5.4. The Inadequacy of Current PAH Management

The current consensus-based practice recommendations for the management of patients with PAH were firstly established by the European Society of Cardiology (ESC) and the European Respiratory Society (ERS) and later renovated in 2018 to include new evidence [42]. Therefrom, an initial combination therapy, instead of monotherapies, becomes the standard approach in newly diagnosed classic PAH patients without significant cardiopulmonary comorbidities. For instance, oral combination therapy with endothelin receptor antagonists and PDE type 5 inhibitors, or soluble guanylate cyclase stimulators, respectively, are recommended to PAH patients with a low or intermediate risk based on the risk assessment in the European guidelines. For patients who are assessed to be high risk, triple combination therapy, including a subcutaneous or intravenous prostacyclin analogue, should be deliberately considered. Furthermore, as reported by clinical trials, improvement in patient outcomes also demonstrates the necessity of targeting multiple pathways in PAH via the use of combination drug therapy [43]. Despite the improvement in functional capacity and hemodynamics, as well as a reduction in hospital admissions by medical therapy, the aforementioned vasodilating agents are extremely costly yet still not curative as they do not target key features of PAH pathogenesis and cannot significantly decrease mortality [18]. During the past two decades, more than ten drugs have been developed and approved for the treatment of PAH. However, only intravenous epoprostenol was reported to be able to reduce mortality [11]. When pharmacological treatment fails, surgical intervention only by lung transplantation becomes the last resort, which unfortunately is often unavailable to many patients due to a dearth of organ donors. Therefore, new promising targets in treating PAH are in urgent need.

## 6. Targets under Consideration

### 6.1. Platelet-Derived Growth Factor Receptor

Given the fact that the PDGF is the predominant trigger in PASMCs’ proliferation and migration [16], it is not surprising that using receptor tyrosine kinase inhibitors, such as imatinib against the PDGF receptor (PDGFR), has been regarded as an attractive strategy to treat PAH. Imatinib was reported to not only reverse experimental pulmonary hypertension in the rat disease model, but also suggested having hemodynamic improvements in patients with end-stage PAH [44], although the side effects are due to a lack of selective kinase inhibition narrow the clinical utility of imatinib. Similarly, the Phase II studies initiated by Novartis of nilotinib, a non-selective PDGFR, were terminated due to the reveal of significant cardiovascular adverse events [45].

### 6.2. PI3K/AKT Signaling Cascades

Aberrant cell migration and excessive proliferation of PASMCs are the critical pathological features of PAH; targeting cell migration and proliferation has been widely recognized as a therapeutic strategy against PAH. Among these, the published literature highlights the importance of the phosphoinositide 3-kinases (PI3K)-AKT signaling cascade in the pathological development of PAH. The PI3K/AKT pathway has been well disclosed to contribute to cell proliferation and cell survival in various cell types, including PASMCs [46]. Suppressing the PI3K/AKT by using the small molecular inhibitor, LY294002, has been previously demonstrated to reverse hypoxia-induced anti-apoptotic PASMCs proliferation in response to hypoxic conditions [47]. Pharmacological studies of multi-kinase inhibitors, such as sorafenib, were also evidenced to show therapeutic benefits in reducing and reversing the progress of MCT-induced PAH in rat models [48]. The knockout of the Akt1 gene in mice was also reported to attenuate pulmonary vascular remodeling and to reduce the wall thickness of the pulmonary artery, which reflects its protective effect against the disease course of hypoxia-induced pulmonary hypertension [49]. Furthermore, the mammalian target of rapamycin (mTOR), one of the major downstream effectors of the PI3K/AKT pathway, which implicates cell proliferation, migration, differentiation, and protein synthesis, was also examined as a target in PAH therapeutics. Strong evidence has highlighted the pathogenic role of mTOR in promoting PASMCs’ proliferation and pulmonary vascular remodeling in both clinical and experimental PAH models [50]. Attempting to treat PAH by using the mTOR inhibitor rapamycin can be traced back to 2001. Administration with rapamycin at the dose of 1–5 mg/kg/day exhibited a significant inhibitory effect on the development of MCT-induced PAH in both prevention and reversal models, and the results were more potent than that treated with imatinib [51]. This result suggests that mTOR could be the therapeutic target for developing a strategy against PAH if a more specific and selective drug is to be used.

### 6.3. Regulators in Glycolytic Metabolism

Notably, a cancer-like transformed glucose metabolism has recently been noticed as a feature in PAH pathogenesis. A glycolytic gene overexpression has been discovered in the pulmonary artery fibroblasts of PAH patients [52]. The treatment of PAH, thus, implicates the importance of targeting the main regulators of glucose uptake as glycolysis-related signaling pathways may be the key to suppressing the development of PAH. For instance, dichloroacetate (DCA), an inhibitor of the pyruvate dehydrogenase kinase, has been approved to activate pyruvate dehydrogenase (PDH, a gatekeeping enzyme of glucose oxidation) and increase mitochondrial respiration, which further reduces the mean PA pressure and pulmonary vascular resistance and improves the functional capacity in idiopathic PAH patients [37]. Similarly, studies revealed that the activity of hexokinase 2 (HK-2) is significantly increased in abnormally proliferating cells rather than in normal cells [53]. HK-2 plays a first rate-limiting role in glycolysis for energy supply in these rapid-growing cells. Therefore, using the non-coding micro-RNA, miR125a-5p, to target HK-2 was also reported to inhibit glycolysis, which further improves PAH [54].

### 6.4. Heat Shock Protein (HSP) 90

Recently, consideration of targeting heat shock protein 90 (HSP90), an essential molecular chaperone, as a therapeutic strategy against PAH has arisen [55]. HSP90 was observed to be upregulated in both the plasma and membrane walls of pulmonary arterioles in PAH patients [56]. The level of HSP90 has been reported to be positively correlated to not only cell proliferation under stress conditions but also the mean pulmonary arterial pressure, therefore implying this ubiquitous chaperone protein is also involved in PAH pathogenesis [56,57]. Using 17-AAG, an HSP90-inhibitor, was evidenced to alleviate an MCT-induced PAH in vivo by improving pulmonary arteriole remodeling [56]. Intriguingly, Boucherat et al. reported that mitochondrial, but not cytosolic HSP90 accumulation, promotes cell survival and vascular remodeling in PAH-PASMCs [57]. This suggests that mitochondria may play a more critical role in PAH pathogenesis than what we have already known. According to their report, selectively inhibiting mitochondrial HSP90 by using gamitrinib, a mitochondrial matrix inhibitor, was demonstrated to reduce cell proliferation and resistance to apoptosis in PAH-PASMCs in vitro [57]. In an experimental MCT-induced PAH rat model, the gamitrinib has also been confirmed to improve PAH in vivo [57].

### 6.5. High-Mobility Group Box-1 (HMGB1)

Aberrantly elevated high-mobility group box-1 (HMGB1), a critical inflammatory cytokine, with down-regulated BMPR2 and dysregulated mitochondrial fission due to the excessive activation of the GTPase dynamin-related protein 1 (Drp1) have been observed in PAH patients [58,59]. While the in vitro incubation of HMGB1 triggers significant cell proliferation and migration in PASMCs, using the HMGB1 inhibitors, saquinavir and glycyrrhizn, can restore these pathogenic PAH phenotypes [59]. Feng et al. have further demonstrated that HMGB1 activated ERK1/2 to phosphorylate Drp1 and, subsequently, triggered autophagy, which resulted in the lysosomal degradation of BMPR2, and, eventually, induced cell proliferation and migration in PASMCs [58]. The pharmacological inhibition of HMGB1 by glycyrrhizin, the suppression of the ERK1/2 activity, and the knockdown of Drp1, or its disturbed autophagy by chloroquine, either reversed the HMGB1-induced PASMCs’ proliferation/migration in vitro or prevented the MCT-induced PAH development in animals [58]. This may also explain why liraglutide, a drug in diabetic treatment, exhibited a therapeutic consequence in PAH, as aforementioned [29,30]. Altogether, these studies emphatically indicate that targeting HMGB1 and its downstream regulators could be a novel therapeutic strategy in PAH treatments.

### 6.6. Bromodomain and Extra-Terminal (BET) Proteins

A bromodomain and extra-terminal (BET) protein binds acetylated lysine residues on histone via its two N-terminal bromodomains (BD1 and BD2) with an extra-C terminal domain to recruit transcriptional activators for the initiation of transcription [60]. Therefore, using BET inhibitors to disrupt the initial binding of BET proteins to acetylated histones and arrest the transcriptional cascade of oncogenes has been utilized in cancer therapy. The disruption of BET binding significantly reduces cell proliferation and triggers apoptosis in various hematologic malignancies [60]. Recently, bromodomain-containing protein-4 (BRD4), one of the four conserved mammalian BET members, was identified to be increased in PAH pathogenesis [61]. A multicenter validation of using RVX208, a clinically available BET inhibitor, was demonstrated to reveal significant therapeutic benefits in PAH treatment. The administration of RVX208 not only reversed cell hyperproliferation but also improved pulmonary hemodynamics and decreased vascular remodeling in both hypoxia- and MCT-induced PAH models in vivo [62]. Furthermore, an oral administration of RVX208 (Apabetalone) is currently undergoing a phase one: https://clinicaltrials.gov/ct2/show/NCT03655704 (accessed on 14 May 2022) and a phase two: https://clinicaltrials.gov/ct2/show/NCT04915300 (accessed on 14 May 2022) clinical trial to investigate the efficacy and safety of targeting BRD4 in PAH therapy.

### 6.7. p53

p53 is a tumor suppressor gene that widely participates in various cellular regulations, including cell cycle coordination, the activation of DNA repair when DNA has sustained damages, and the initiation of apoptosis, should the damage become irreparable. Since p53 exhibits activity in the growth-suppressive and pro-apoptotic effects in cancer, the role of p53 in cancer-like PAH pathogenesis has been noticed. p53 functions as a transcription factor and was recently observed to be associated with PAH-related DNA damage in endothelial dysfunction [63]. Disturbed p53 function was noted in pulmonary artery endothelial cells in clinical PAH, and an experimental murine hypoxia-induced PH model also revealed that the genetic depletion of p53 resulted in more severe lung manifestations [42,64]. Silencing p53 in PASMCs has also been documented to result in a cancer-like proliferative phenotype with enhanced glycolysis and reduced mitochondrial respiration [53]. A chronic administration of pifithrin-α (PFT, an inhibitor of p53 activity) is sufficient to trigger PAH development in rat models [65]. Furthermore, p53 was reported to be able to activate HSP90 via Aha1, the activator of HSP90, and suppress the PDGFR [66,67]. It is expected, therefore, that the pharmacological induction of the p53 expression/activity by using CP-31398, a p53 stabilizing agent, may be another approach to treat PAH. The potential targets in PAH-targeted therapy and their clinical significances or therapeutic benefits are displayed in Table 2.

## 7. Conclusions

**Figure 3:**
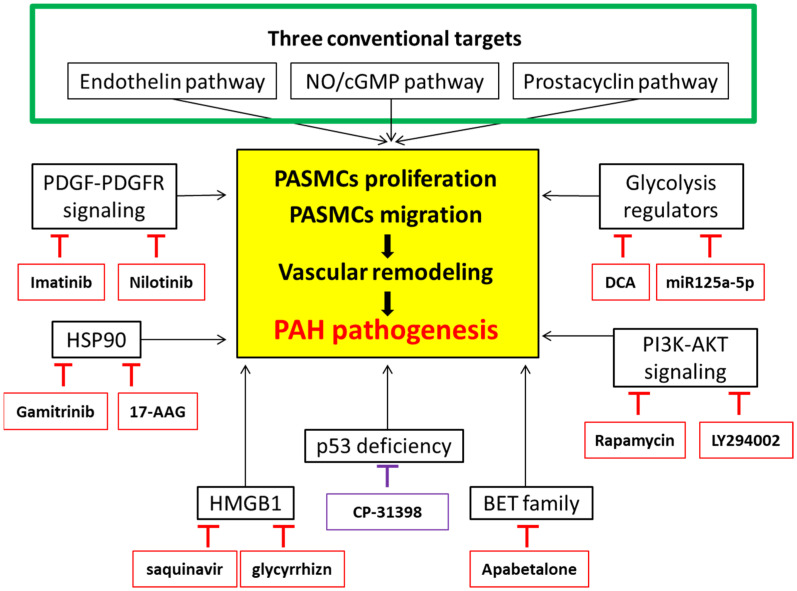
**The graphic summary of the pathogenesis of PAH and the potential therapeutic targets in treating PAH.** Three major cellular signaling pathways have been conventionally identified for targeted therapy in pulmonary arterial hypertension (PAH): (1) The endothelin pathway, (2) nitric oxide (NO)/cyclic guanosine, the monophosphate (cGMP) pathway, and (3) the prostacyclin pathway. In combination, abnormalities in these pathways contribute to PAH pathogenesis caused by vascular remodeling as a result of the proliferation and migration of pulmonary arterial smooth muscle cells (PASMCs). Receptor tyrosine kinase inhibitors (Imatinib, Nilotinib) are used to reduce the effect of platelet-derived growth factor receptors (PDGFR), which are the predominant triggers of unregulated PASMCs’ proliferation and migration, leading to vascular remodeling. 17-AAG, a heat shock protein 90 (HSP90)-inhibitor, and gamitrinib, a mitochondrial matrix inhibitor, can be used to downregulate HSP90, thereby reducing cell proliferation. Saquinavir and glycyrrhizn can also decrease cell proliferation and migration in PASMCs by inhibiting the high-mobility group box-1 (HMGB1). Pharmacological induction of the p53 expression/activity by using a p53 stabilizing agent, CP-31398, can also counteract the effect of p53 silencing or deficiency. Apabetalone, the orally available bromodomain and extra-terminal motif (BET) inhibitor, blocks the binding between acetylation on chromatin histones and BET proteins, which normalizes hemodynamics parameters and pulmonary vascular remodeling in PAH. Suppressing PI3K/AKT by using the small molecular inhibitor, LY294002, may also reverse hypoxia-induced anti-apoptotic PASMCs’ proliferation, as well as inhibit the mammalian target of rapamycin (mTOR), one of the major downstream effectors of the PI3K/AKT pathway, via rapamycin. Dichloroacetate (DCA) and the non-coding micro-RNA, miR125a-5p, targeting hexokinase 2, can decrease glycolysis while increasing mitochondrial respiration.

Understanding the pathogenesis of PAH and the underlying pathways that dictate the progression of this disease is of paramount importance to designing clinical studies in search of an effective remedy. In this review, we surveyed the current literature investigating PAH pathogenesis and the therapeutic management of PAH. We conducted an updated literature review to summarize and discuss the potential therapeutic targets to be employed against PAH, including PDGF/PDGFR signaling, the PI3K/AKT pathway, regulators in glycolytic metabolism, HSP90, HMGB1, BET proteins, and p53. In addition to the three major conventional pathways already used in PAH therapy, targeting PDGF/PDGFR signaling, regulators in glycolytic metabolism, PI3K/AKT pathways, mitochondrial HSP90, HMGB1, and BET proteins by using their specific inhibitors, or a pharmacological induction of the p53 expression, could be attractive strategies for treating PAH (Figure 3). These treatments not only exhibited antiproliferative effects in vitro, but also revealed a clinical therapeutic potential in reversing PAH-caused pulmonary vascular remodeling and normalizing the hemodynamic parameters. We suppose that using clinical drugs, such as liraglutide and apabetalone, which are currently applied in other diseases, such as cancers and diabetes, but yet irrelevant to PAH treatments, might be considered in PAH therapeutics, at least as a part of combination therapy if the targets of the drug are overlapped and involved in PAH pathogenesis. Hopefully, any efforts made along the way would be beneficial for developing future strategies to overcome the still insurmountable clinical challenges posed by this deadly disorder.

## Figures and Tables

**Table 1:** Similarities between PASMCs in PAH pathogenesis and cancer cells.

	PASMCs in PAH	Cancer Cells
**Cell proliferation**	↑	↑
**Cell migration**	↑	↑
**Cell apoptosis**	↓	↓
**Glycolysis**	↑	↑
**Fatty acid synthesis**	↑	↑
**Glutamine metabolism**	↑	↑
**Mitochondrial respiration**	↓	↓

**Table 2:** The potential targets in PAH-targeted therapy and their clinical significances. (Numbers in parentheses denote the reference numbers).

Target	Drug	Clinical Significance/Therapeutic Benefits
PDGF receptor [44,45]	Imatinib	RVSP↓ PAP↓ RV hypertrophy↓ Pulmonary vascular remodeling↓
	Nilotinib	Terminated due to severe adverse events
PI3K/AKT pathway [47,48]	LY294002	Cell proliferation↓
	Sorafenib	RVSP↓ RV hypertrophy↓ Pulmonary vascular remodeling↓ Cell proliferation↓ Cell apoptosis↑
	Rapamycin	PAP↓ RVSP↓ RV hypertrophy↓ Pulmonary vascular remodeling↓ Cell proliferation↓
Glycolytic metabolism [53,54]	Dichloroacetate	Mitochondrial respiration↑ mPAP↓ PVR↓
	miR125a-5p	MCT-induced PASMCs glycolysis↓ MCT-induced PASMCs proliferation↓ RV hypertrophy↓ mPAP↓
HSP90 [56,57]	17-AAG	Pulmonary vascular remodeling↓ Cell proliferation↓ Cell migration↓
	Gamitrinib	Cell proliferation↓ Cell apoptosis↑ mPAP↓ RVSP↓ Pulmonary vascular remodeling↓
HMGB1 [58,59]	Saquinavir	Hemodynamic parameters↓ Pulmonary vascular remodeling↓
	Glycyrrhizn	Hemodynamic parameters↓ Pulmonary vascular remodeling↓
BET proteins [61,62]	Apabetalone (RVX208)	Cell proliferation↓ Cell apoptosis↑ Hemodynamics parameters↓ Pulmonary vascular remodeling↓
p53 activation [66]	CP-31398	HSP90 activity↓

## Data Availability

Not applicable.
